# Improvements across a range of patient-reported domains with fremanezumab treatment: results from a patient survey study

**DOI:** 10.1186/s10194-020-01177-4

**Published:** 2020-09-04

**Authors:** Dawn C. Buse, Sanjay K. Gandhi, Joshua M. Cohen, Verena Ramirez-Campos, Blaine Cloud, Ronghua Yang, Robert P. Cowan

**Affiliations:** 1 Albert Einstein College of Medicine, Bronx, NY 10461 USA; 2 Teva Branded Pharmaceutical Products R&D, Inc., West Chester, PA USA; 3 Clinical SCORE, Chadds Ford, PA USA; 4 Stanford University, Stanford, CA USA

**Keywords:** Migraine, Prevention, Satisfaction, Treatment preference, Sleep, Anxiety

## Abstract

**Background:**

The long-term safety and efficacy of fremanezumab were evaluated in a 52-week extension study (NCT02638103). Patient satisfaction with fremanezumab, dosing preferences, and patient-reported outcomes were assessed in a subpopulation who completed the extension study and consented to a follow-up questionnaire.

**Methods:**

In the extension study (*N* = 1842), adults with migraine were randomized to quarterly or monthly fremanezumab. After completing active treatment, patients answered a survey evaluating patient satisfaction, treatment and dosing preferences, and changes in patient-reported outcomes.

**Results:**

Of the 557 patients who could have been contacted upon completing the extension study, 302 consented and 253 completed the survey. The mean (standard deviation) satisfaction rating for fremanezumab was 6.1 (1.4; 1 = “extremely dissatisfied” to 7 = “extremely satisfied”). Most patients (175 [69.2%]) preferred quarterly over monthly fremanezumab dosing. Among patients taking antiepileptics (most common class of prior preventive medication; *n* = 130), 91.5% preferred fremanezumab. Patients reported improvements in anxiety (74 [67.9%]), sleep quality (143 [56.5%]), and quality of time spent with others (210 [83.0%]) with fremanezumab.

**Conclusion:**

In this study, treatment satisfaction with fremanezumab was high, most patients preferred quarterly fremanezumab dosing, and fremanezumab was generally preferred to prior preventive medications.

**Trial registration:**

ClinicalTrials.gov NCT02638103 (HALO LTS), registered December 22, 2015.

## Background

Multiple treatment guidelines and consensus statements suggest that preventive treatment should be considered for patients with migraine who experience frequent attacks (≥4 migraine days per month), experience associated disability, or overuse or experience poor tolerability to acute treatments [1–4]. In a recent analysis of US population–based respondents with migraine in the Migraine in America Symptoms and Treatments (MAST) Study, approximately 63% of patients reported mild or greater migraine-related disability; however, only 14.5% of patients with migraine reported using a preventive medication [5]. These rates are similar to findings reported a decade earlier in the American Migraine Prevalence and Prevention (AMPP) Study, where only 12.4% of people with migraine indicated that they were taking a preventive medication for their migraine, despite approximately 32% being candidates for preventive therapy [2, 6].

Adherence with traditional migraine preventive therapies is generally low, with approximately 77% to 85% of patients discontinuing their initial migraine preventive medication within 1 year [7, 8]. Patients who discontinue or switch preventive treatments experience a substantial burden in terms of impaired function and quality of life [9], as well as increased healthcare resource utilization and costs [7]. The most common reasons for discontinuation include lack of efficacy and poor tolerability [10]. Thus, it may be of value to healthcare professionals to assess patient satisfaction and perceptions of treatment characteristics in order to potentially improve adherence and optimize clinical outcomes. Monoclonal antibodies (mAbs) targeting the calcitonin gene-related peptide (CGRP) pathway may offer better-tolerated, efficacious alternatives to traditional migraine pharmacologic preventive therapies, none of which were developed specifically for migraine [11, 12].

Fremanezumab is a fully humanized mAb (IgG2Δa) that selectively targets the CGRP-ligand [13] and has been approved in the United States for the preventive treatment of migraine in adults and in Europe for migraine prophylaxis in adults who have ≥4 migraine days per month [14, 15]. The efficacy of fremanezumab for the preventive treatment of migraine has been demonstrated in the HALO and FOCUS clinical trials in patients with episodic migraine (EM) or chronic migraine (CM), even in those with inadequate response to up to four prior migraine preventive medication classes [16–18]. The long-term safety and efficacy of fremanezumab have also been evaluated in a 52-week extension study [19].

Following the 52-week fremanezumab extension study, a survey was conducted to evaluate participant satisfaction with fremanezumab as a migraine preventive treatment, the perceived value of dosing flexibility (including perceived impact on adherence), the impact of fremanezumab on the burden of migraine, and preference for fremanezumab versus other prior preventive treatments. Here, we report the results of survey responses from 253 patients (response rate = 45.4%) who completed the 52-week extension study and responded to the survey questions.

## Methods

### Participants and extension study dosing

In the 52-week fremanezumab extension study (ClinicalTrials.gov registration number: NCT02638103), 1842 adults with EM or CM were randomized to receive quarterly or monthly fremanezumab. A total of 1439 (78.1%) patients completed the 52-week study.

An online survey was administered to adult patients over 18 years of age who had completed the extension study. For the survey, patients were recruited through the clinical trial sites based in the United States from the extension study. A total of 41 of the available 83 extension study sites contacted their patients to ask them to voluntarily consent to take a survey. After consenting to participate, patients were directed to an online web survey. Patients who started another CGRP pathway–targeted migraine treatment after the extension study were excluded from participating in the survey. In total, 557 patients could have been invited via email to complete the survey, and 302 patients consented. Of the 302 patients who consented, 49 did not meet the study criteria (ie, had taken a CGRP pathway–targeted treatment after completing the extension study) and were excluded. Thus, a subpopulation of 253 eligible participants responded to the survey 1 to 24 months after the last extension study visit (Supplementary Figure 1). The survey, informed consent document, and all other relevant patient materials were approved by a central institutional review board (Quorum).

Full details of the patient enrollment criteria for the 52-week extension study have been reported previously [19]. Briefly, that study included adult patients with EM or CM who had completed treatment in the pivotal randomized, double-blind, placebo-controlled phase 3 HALO EM (NCT02629861) and HALO CM (NCT02621931) studies of fremanezumab, as well as new patients who did not roll over from the phase 3 studies. During the extension study, all patients were blinded to their treatment assignment. Patients were randomized 1:1 to either quarterly fremanezumab (675 mg) or monthly fremanezumab (225 mg) for 52 weeks of treatment, with placebo injections used to match the number of doses between the two dosing regimens to maintain the blind. Patients with CM who were newly enrolled (ie, not rollover patients from the parent HALO studies) or patients receiving placebo in the parent HALO studies received a starting dose of 675 mg of fremanezumab in the monthly arm.

### Study assessments

Participants completed an online patient experience survey, which took approximately 20 to 40 min, at 1 to 24 months after their last study visit in the 52-week extension study. Median time between the last trial visit and survey was 11 months. This survey included 31 questions (developed specifically for this study) assessing participant satisfaction overall and across different aspects of treatment or treatment dimensions; participant preference for and satisfaction with fremanezumab; dosing preference and the value of dosing flexibility; and perceived changes from the 3-month baseline period before the first injection in anxiety, depressed mood, sleep, acute medication use, functioning, and work performance while taking the study medication. Changes in anxiety and depressed mood were only assessed in participants reporting those respective psychological symptoms during the baseline period. The majority of survey questions were constructed with Likert-type response scales consisting of either 7- or 11-point scales. Treatment satisfaction questions were generally rated using a 7-point scale, where 1 = “extremely dissatisfied” and 7 = “extremely satisfied.” Life impact, psychological functioning, sleep, and over-the-counter/rescue medication changes were generally rated using an 11-point scale where 0 = “significantly worse (less),” 5 = “no difference,” and 10 = “significantly better (more).” The survey question regarding acute medication use was a single-perception item comparing use from the 3-month baseline period before the first injection of study drug with use during study treatment. Due to an error in question wording to assess perceived changes in depressed mood from the 3-month baseline period before the first injection, participants were re-surveyed with corrected question wording. The results for depressed mood are thus reported only for the re-surveyed sub-sample of participants (*n* = 68).

### Statistical analyses

The anticipated sample size for this survey was 208 participants from the 52-week extension study, based on an expected consent rate of 25% to 30% of patients from 50% of participating clinical trial sites. Categorical response data were summarized as numbers and percentages of patients reporting each possible response. Data reported on a 7- or 11-point Likert scale were also summarized using descriptive statistics (mean and standard deviation [SD]). Results were reported for the overall sample taking the survey, as well as stratified by migraine classification (EM or CM) and fremanezumab dosing group (quarterly or monthly).

## Results

### Participants

In the population who completed the survey (*n* = 253), 131 patients had been randomized to quarterly fremanezumab, and 122 patients had been randomized to monthly fremanezumab in the extension study. A total of 119 patients had EM, and 134 patients had CM. All patients who completed the survey had received active treatment during the extension study; 134 patients had also received fremanezumab during prior phase 3 trials (HALO EM and HALO CM).

The majority of participants were women (88.5% [224/253]), and the average age was approximately 46 years. Demographic and baseline characteristics were well-balanced across migraine classifications and treatment groups for this survey population and were consistent with the demographic and baseline characteristics of the overall extension study population (Table 1). Over half (57.3% [145/253]) of participants had tried at least one migraine preventive medication before entering fremanezumab clinical trials, with antiepileptics (51.4% [130/253]) and antihypertensives (24.5% [62/253]) being the most commonly used preventive classes (Supplementary Figure 2). Among 557 potentially eligible participants, those who did (*n* = 253) and those who did not participate (*n* = 304) did not differ in age, gender, or headache frequency distribution.

**Table 1:** Baseline and Demographic Characteristics of the Survey Population^a^

**Characteristic**	**Total (*n*** **= 253)**	**EM (*n*** **= 119)**	**CM (*n*** **= 134)**	**Quarterly (*n*** **= 131)**	**Monthly (*n*** **= 122)**
Mean (SD) age, years	45.5 (11.6)	46.9 (12.3)	45.2 (11.0)	47.3 (10.9)	44.7 (12.2)
Mean (SD) age at migraine diagnosis, years	23.5 (9.4)	24.5 (10.4)	22.5 (8.4)	24.6 (10.0)	22.3 (8.7)
Sex, n (%)					
Female	224 (88.5)	104 (87.4)	120 (89.6)	120 (91.6)	104 (85.2)
Male	29 (11.5)	15 (12.6)	14 (10.4)	11 (8.4)	18 (14.8)
Race, n (%)^b^					
Caucasian	210 (83.0)	99 (83.2)	111 (82.8)	103 (78.6)	107 (87.7)
Black/African American	24 (9.5)	13 (10.9)	11 (8.2)	16 (12.2)	8 (6.6)
Asian/Pacific Islander	8 (3.2)	2 (1.7)	6 (4.5)	4 (3.1)	4 (3.3)
Native American	1 (0.4)	1 (0.8)	0	1 (0.8)	0
Other	7 (2.8)	4 (3.4)	3 (2.2)	4 (3.1)	3 (2.5)

*CGRP* calcitonin gene-related peptide, *CM* chronic migraine, *EM* episodic migraine, *SD* standard deviation

^a^Baseline and demographic data are shown for the 253 eligible patients who completed the survey. Data are not shown for the 49 patients who consented but did not meet the study criteria (ie, took a CGRP pathway–targeted treatment after completing the extension study) and were excluded from participation in the survey study

^b^3 patients did not provide a response

### Patient satisfaction

The mean (SD) overall satisfaction rating for fremanezumab (possible response, 1 to 7, with higher scores indicating greater satisfaction) was 6.1 (1.4). The satisfaction ratings for fremanezumab across evaluated aspects of treatment (ability to prevent or treat migraine, way the medication relieved symptoms, time it took to start working, ease of medication administration) were consistently high, with mean satisfaction ratings ranging from 5.7 to 6.1 for the overall population (Table 2). In the overall population, 226 (89.3%) patients reported high satisfaction (ratings of 5 to 7) with fremanezumab treatment, regardless of migraine classification (EM, 93.3% [111/119]; CM, 85.8% [115/134]) and dosing group (quarterly, 90.1% [118/131]; monthly, 88.5% [108/122]). High proportions of patients, over half in each of the EM and CM and quarterly and monthly dosing regimen subgroups, also reported satisfaction across the evaluated dimensions of treatment (Supplementary Figure 3).

**Table 2:** Patient Treatment Satisfaction Scores With Fremanezumab on the Likert Scale^a,b,c^

**Efficacy dimension, mean (SD)**	**Overall (*n*** **= 253)**	**EM (*n*** **= 119)**	**CM (*n*** **= 134)**	**Monthly (*n*** **= 122)**	**Quarterly (*n*** **= 131)**
Ability to prevent or treat migraine	6.1 (1.4)	6.5 (0.9)	5.8 (1.6)	6.2 (1.5)	6.1 (1.3)
Way the medication relieved symptoms	6.1 (1.4)	6.4 (1.1)	5.8 (1.6)	6.1 (1.5)	6.1 (1.3)
Time it took to start working	5.7 (1.4)	5.9 (1.3)	5.5 (1.5)	5.7 (1.5)	5.7 (1.4)
Ease of medication administration	6.0 (1.4)	6.1 (1.3)	5.9 (1.4)	6.1 (1.2)	5.9 (1.5)

CM, chronic migraine; EM, episodic migraine

^a^For ability to prevent or treat migraine, way the medication relieved symptoms, and time it took to start working, patients responded to the following questions: “How satisfied or dissatisfied were you with…”

• “…the ability of the clinical trial medication to prevent or treat your migraine?”

• “…the amount of time it took the clinical trial medication to start working?”

• “…the way the clinical trial medication relieved your migraine symptoms?”

^b^For ease of medication administration, patients responded to the following question: “How easy or difficult was it to have the clinical trial medication administered in the clinical trial site (doctor’s office)?”

^c^The Likert Scale ranged from: 1 = “extremely dissatisfied” to 7 = “extremely satisfied”; or 1 = “extremely difficult” to 7 = “extremely easy” (for ease of medication administration question only)

**Fig. 1:**
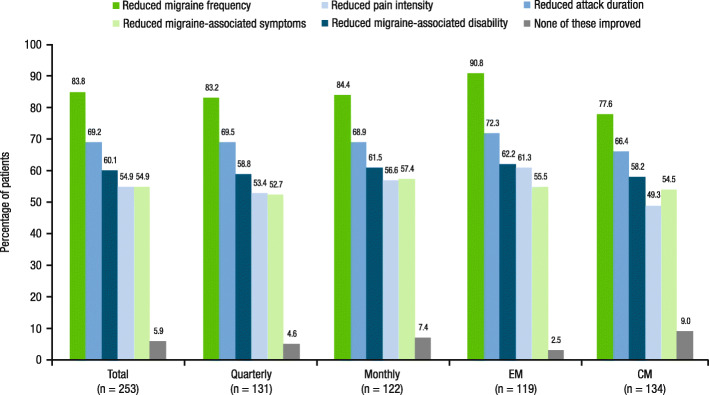
Patient satisfaction with efficacy dimensions of fremanezumab^a^. CM, chronic migraine; EM, episodic migraine. ^a^Patients responded to the following question: “Thinking specifically of the experimental medicine’s ability to prevent migraine, with which dimension of your migraine relief were you satisfied? *Select all that apply*.” This question had the following response options: “reducing attack frequency,” “reducing migraine pain intensity,” “reducing attack duration,” “reducing migraine-associated symptoms (like nausea, light & sound sensitivity),” “reducing migraine associated disability (ability to work and participate in activities),” and “none of the above”

Across efficacy dimensions, patients in the total population most commonly reported satisfaction with reduced migraine frequency with fremanezumab (83.8% [212/253]; Fig. 1). Patients also most commonly reported satisfaction with reduced migraine frequency with fremanezumab in the EM (90.8% [108/119]) and CM (77.6% [104/134]) subgroups and across the quarterly (83.2% [109/131]) and monthly (84.4% [103/122]) dosing regimens. For the remaining efficacy dimensions (reduced migraine-associated symptoms, reduced pain intensity, reduced migraine-associated disability, reduced attack duration), between 54.9% (139/253) and 69.2% (175/253) of patients in the total population reported satisfaction (Fig. 1).

### Dosing preference and value of dosing flexibility

**Fig. 2:**
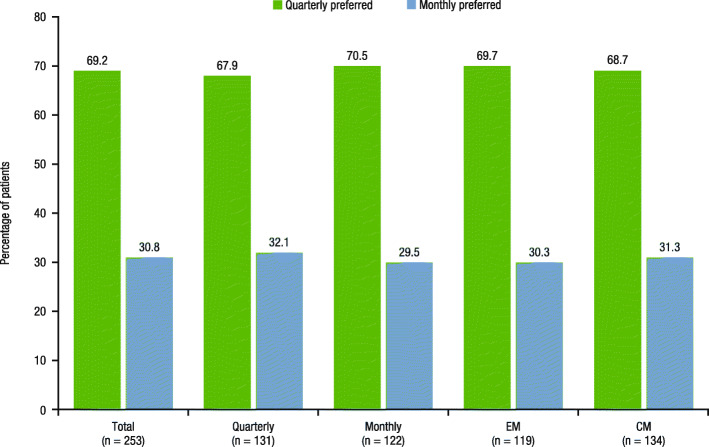
Patient-reported preference for quarterly or monthly fremanezumab dosing regimen^a^. CM, chronic migraine; EM, episodic migraine. ^a^Patients responded to the following question: “If the effectiveness was similar no matter which option you choose, would you rather take the injectable migraine medicine once a month, or once every 3 months?” This question had the following response options: “monthly” or “once every 3 months”

**Fig. 3:**
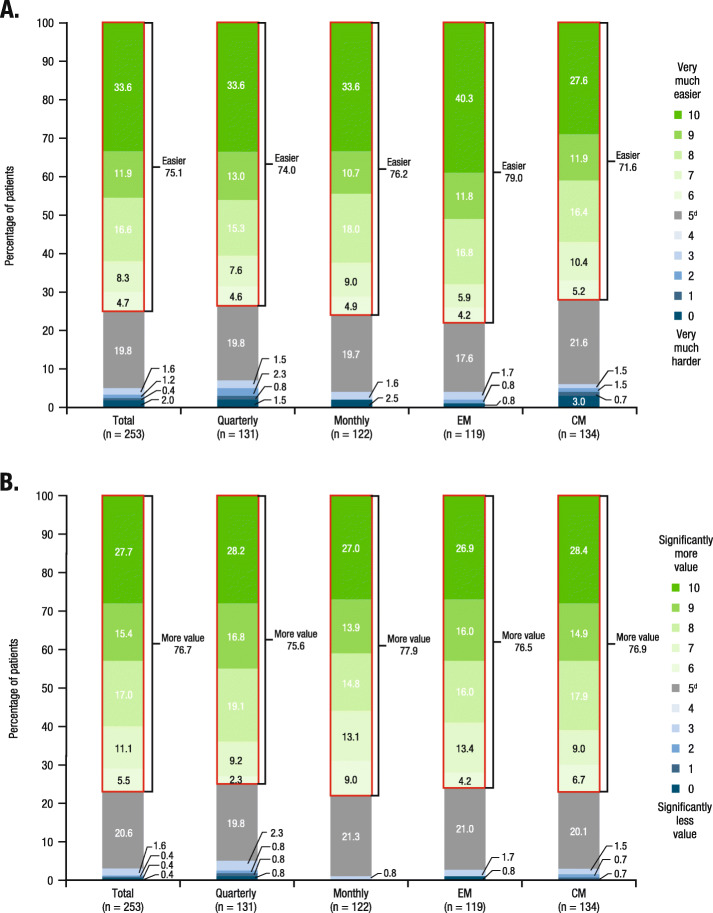
Patient perceptions of dosing flexibility: **a** impact on ease of use^a^; **b** value^b,c^. CM, chronic migraine; EM, episodic migraine. ^a^For patient perceptions of the impact of dosing flexibility on the ease of taking a migraine preventive treatment as prescribed, patients responded to the following question: “We want to learn from you the importance of having a choice of dosing frequency for this migraine medicine. The dosing frequencies are either monthly or every-3-months. On a scale of 0 to 10, where 0 is ‘very much harder’ and 10 is ‘very much easier,’ how much harder or easier would it be to take your medicine regularly, as prescribed by your doctor, if you had the flexibility to select either monthly or every-3-month injections, based on your preference after discussion with your doctor?” ^b^For patient perceptions on the value of having dosing flexibility to allow taking the injection quarterly or monthly, patients responded to the following question: “On a scale of 0 to 10, where 0 is ‘significantly less value’ and 10 is ‘much more value,’ how much more valuable to you is the flexibility of taking your injection either once a month or once every 3 months after consulting with your doctor?” ^c^Percentages may not total 100%. ^d^No difference

The majority of patients overall (69.2% [175/253]) preferred quarterly dosing, regardless of dosing regimen received during the extension study (quarterly, 67.9% [89/131]; monthly, 70.5% [86/122]) or migraine classification (EM, 69.7% [83/119]; CM, 68.7% [92/134]; Fig. 2). A total of 75.1% (190/253) of patients endorsed that having dosing flexibility makes it easier to adhere to this migraine preventive medication (Fig. 3a). The proportion of patients reporting that dosing flexibility would make taking migraine preventive treatment “very much easier” (maximum score) was higher among patients with EM (40.3% [48/119]) versus patients with CM (27.6% [37/134]), while 33.6% reported a rating of “very much easier” in both the quarterly (44/131) and monthly dosing groups (41/122). In the total population, 76.7% (194/253) of patients believed that having dosing flexibility added value to the migraine treatment, with a similar proportion of patients reporting this perception in the EM (76.5% [91/119]) and CM (76.9% [103/134]) subgroups, as well as in the quarterly (75.6% [99/131]) and monthly (77.9% [95/122]) dosing groups (Fig. 3b). The proportion of patients reporting a maximum score of 10 (“significantly more value”) was similar across treatment groups (quarterly, 28.2% [37/131]; monthly, 27.0% [33/122]) and migraine classification subgroups (EM, 26.9% [32/119]; CM, 28.4% [38/134]).

### Patient preference and satisfaction with Fremanezumab

Treatment satisfaction scores (possible score, 1 to 7, with higher scores indicating greater satisfaction) were higher with fremanezumab (mean, 6.1) than with any other prior migraine preventive medication class (mean, 2.7 to 3.0; Supplementary Figure 4). Regardless of prior migraine preventive medication class used, over three-quarters of patients in each medication class reported a preference for fremanezumab versus prior medication (Table 3). In the overall population, the proportion of patients reporting a preference for fremanezumab ranged from 82.1% (23/28) among those previously using onabotulinumtoxinA to 96.3% (26/27) among those previously using selective serotonin reuptake inhibitors (SSRIs) or serotonin-norepinephrine reuptake inhibitors (SNRIs). Of the patients who were previously treated with antiepileptics and antihypertensives, the two most frequently used preventive classes for this patient sample, 91.5% (119/130) and 88.7% (55/62), respectively, expressed a preference for fremanezumab. The top three reasons for preference for fremanezumab treatment were greater reductions in migraine attack frequency, intensity, and duration (Table 3). When patients with prior onabotulinumtoxinA use were asked to compare their injection experience with onabotulinumtoxinA to their injection experience with fremanezumab, 89.3% (25/28) favored the injection experience with fremanezumab (Supplementary Figure 5).

**Table 3:** Reasons Patients Preferred Fremanezumab by Category of Prior Preventive Treatment^a,b^

**Prior preventive treatment**^**c**^	**Antiepileptics** ***N*** **= 130**	**Antihypertensives** ***N*** **= 62**	**Tricyclic antidepressants** ***N*** **= 53**	**OnabotulinumtoxinA** ***N*** **= 28**	**SSRIs/SNRIs** ***N*** **= 27**
**Reason for preventive treatment preference, n (%)**^**d**^	**Preferred fremanezumab** ***n*** **= 119 (91.5%)**	**Preferred fremanezumab** ***n*** **= 55 (88.7%)**	**Preferred fremanezumab** ***n*** **= 49 (92.5%)**	**Preferred fremanezumab** ***n*** **= 23 (82.1%)**	**Preferred fremanezumab** ***n*** **= 26 (96.3%)**
Reduces attack frequency	100 (84.0)	48 (87.3)	40 (81.6)	18 (78.3)	20 (76.9)
Reduces migraine intensity	89 (74.8)	38 (69.1)	38 (77.6)	17 (73.9)	17 (65.4)
Reduces attack duration	81 (68.1)	32 (58.2)	32 (65.3)	14 (60.9)	15 (57.7)
Reduces migraine-associated symptoms	71 (59.7)	27 (49.1)	30 (61.2)	14 (60.9)	15 (57.7)
Reduces migraine-associated disability	74 (62.2)	30 (54.5)	27 (55.1)	14 (60.9)	16 (61.5)
Causes less side effects	78 (65.5)	22 (40.0)	33 (67.3)	8 (34.8)	12 (46.2)
More convenient	45 (37.8)	23 (41.8)	19 (38.8)	8 (34.8)	9 (34.6)
Other	1 (0.8)	0	0	2 (8.7)	2 (7.7)

*SNRI* serotonin-norepinephrine reuptake inhibitor, *SSRI* selective serotonin reuptake inhibitor

^a^Patients may have received prior preventive treatments in more than one class, and patients reported their preference for fremanezumab versus the prior treatment for each prior preventive treatment used. Thus, patients in the survey study sample may have reported preference for fremanezumab versus prior treatment for more than one class of treatment

^b^Patients responded to the following question for each preventive treatment that they reported having taken in the 5 years prior to the clinical trial: “Overall, which medicine did you prefer more, the injectable medicine you received as part of the clinical trial or (*prior migraine preventive medication*)?”

^c^Preferred prior preventive treatment: antiepileptics, 11 (8.5%); antihypertensives, 7 (11.3%); tricyclic antidepressants, 4 (7.5%); onabotulinumtoxinA, 5 (17.9%); SSRI/SNRI, 1 (3.7%)

^d^Percentages were calculated using the number reporting preference for fremanezumab as a denominator

### Self-reported changes in anxiety, depression, and sleep quality

**Fig. 4:**
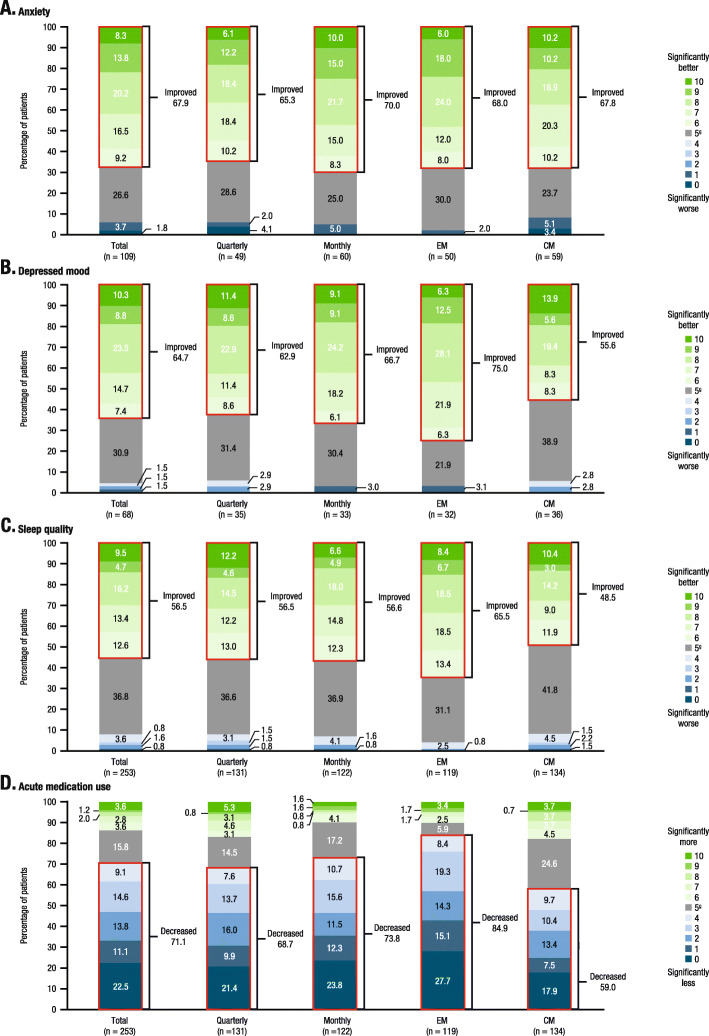
Patient perceptions^a^; **a** anxiety^b,c^; **b** depressed mood^b,d^; **c** sleep quality^b^; **d** acute medication use changes^e,f^. CM, chronic migraine; EM, episodic migraine. ^a^Changes from baseline; 3-month baseline period before the first injection during fremanezumab treatment. ^b^For the changes in anxiety, depressed mood, and sleep quality, patients responded to the following questions: “Compared to the 3-month baseline period before the first injection, on a scale of 0 to 10, where 0 is ‘significantly worse’ and 10 is ‘significantly better’…” “…how much change in anxiety level did you feel while you were taking the study medicine?” “…how much change in depressed mood did you feel while you were taking the study medicine?” “…how much worse or better was your sleep quality while you were taking the study medicine compared to the 3-month baseline period before the first injection?”. ^c^Impact of fremanezumab treatment on anxiety was only evaluated for patients with self-reported anxiety during the 3-month baseline period (n = 109). ^d^Due to a data anomaly, the baseline depression question was re-validated among survey participants. The results here are in a sub-sample of patients with self-reported depressed mood during the baseline period (n = 68). ^e^For the changes in acute medication use, patients responded to the following question: “Compared to the 3-month baseline period before the first injection, on a scale of 0 to 10, where 0 is ‘significantly less’ and 10 is ‘much more,’ how much did you rely on rescue or abortive medicines that stop migraine symptoms (ex: sumatriptan, naratriptan, rizatriptan) and over-the-counter medicines while you were taking the study medicine?” ^f^Percentages may not total 100% due to rounding. ^g^No difference

Of the patients who reported experiencing anxiety during the 3-month baseline period before the first injection (*n* = 109), the majority (67.9% [74/109]) reported improvements in anxiety while taking fremanezumab (Fig. 4a). Similarly, of the patients who reported experiencing depressed mood during the baseline period (*n* = 68), the majority (64.7% [44/68]) reported experiencing improvements in depressed mood while taking fremanezumab (Fig. 4b). In the total survey population, improvements in sleep quality were reported by 56.5% (143/253) of patients during fremanezumab treatment (Fig. 4c). The proportion of patients with baseline anxiety reporting improvements in anxiety during fremanezumab treatment were consistent regardless of migraine classification (EM, 68.0% [34/50]; CM, 67.8% [40/59]) and dosing regimen (quarterly, 65.3% [32/49]; monthly, 70.0% [42/60]) received during the extension study. The proportions of patients with baseline depression reporting improvements in depressed mood were variable across migraine classifications, with 75.0% (24/32) of EM patients and 55.6% (20/36) of CM patients and reporting improvements in depressed mood, as were the proportions of patients in the total population reporting improvements with sleep (EM, 65.5% [78/119] vs CM, 48.5% [65/134]). The proportions of patients with baseline depressed mood who reported improvements in depressed mood during fremanezumab treatment and patients in the overall population reporting improvements with sleep during fremanezumab treatment were comparable across dosing groups (Fig. 4b and c, respectively).

### Self-reported changes in acute medication use

The majority of patients in the total population reported decreases in acute medication use with fremanezumab treatment (71.1% [180/253]; Fig. 4d). Results varied based on migraine classification, with 59.0% (79/134) of CM patients and 84.9% (101/119) of EM patients reporting decreases in acute medication use. Regardless of dosing regimen, more than two-thirds of patients reported a decrease in acute medication use (quarterly, 68.7% [90/131]; monthly, 73.8% [90/122]).

### Self-reported changes in functioning and work performance

**Fig. 5:**
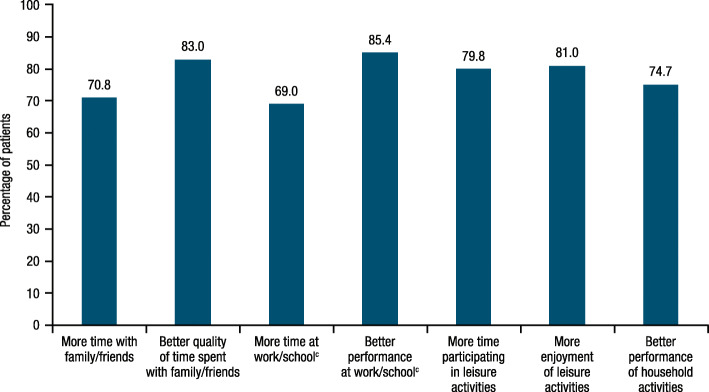
Proportion of patients overall who reported improvements in psychosocial and quality-of-life domains (*n* = 253)^a,b^. ^a^For time with family/friends, quality of time with family/friends, time at work/school, time participating in leisure activities, and enjoyment of leisure activities, patients responded to the following questions: “Compared to the 3-month baseline period before the first injection, on a scale of 0 to 10, where 0 is ‘significantly less’ and 10 is ‘significantly more,’…” “…how much less or more time did you spend with friends and family while you were taking the study medicine?” “…how much change did you experience in the quality of time you spent with your friends and family while you were taking the study medicine?” “…how much less or more were you able to attend work or school while you were taking the study medicine?” “…how much less or more could you participate in leisure and personal activities (i.e., hobbies) while you were taking the study medicine?” “…how much less or more were you able to enjoy leisure and personal activities while you were taking the study medicine?” ^b^For performance at work/school and performance of household activities, patients responded to the following questions: “Compared to the 3-month baseline period before the first injection, on a scale of 0 to 10, where 0 is ‘significantly worse’ and 10 is ‘significantly better,’…” “…how did your work or school performance change while you were taking the study medicine?” “…how much did your ability to perform household activities and chores change while you were taking the study medicine?” ^c^n = 213

When asked about their experience while taking fremanezumab versus the baseline period before the trial, the majority of patients (≥69%) reported improvements and high ratings with treatment (≥7.0; possible response, 0 to 10) across all psychosocial and quality-of-life domains (time and quality of time spent with family/friends, time and performance at work/school, time participating in and enjoyment of leisure activities, performance of household activities; Fig. 5 and Supplementary Figure 6). Although the majority of CM and EM patients reported improvements across all of these domains, relatively higher proportions of EM patients (77.3% [92/119] to 92.4% [110/119] across domains) reported improvements as compared to CM patients (61.7% [71/115] to 85.2% [98/115] across domains), except for the “better quality of time spent with family/friends” domain (EM, 75.6% [90/119]; CM, 89.6% [120/134]; Supplementary Figure 7). Similarly, ratings with treatment across all domains were generally higher for EM patients than for CM patients, except for “better quality of time spent with family/friends” (mean [SD] rating, 7.3 [2.1] vs 7.7 [1.8]) and “better performance at work/school” (7.7 [1.8] vs 7.9 [1.7]; Supplementary Figure 8).

## Discussion

Results of this survey showed that, among patients receiving up to 15 months of fremanezumab treatment, satisfaction with fremanezumab was high overall and across all individual efficacy dimensions. Patients also reported improvements from baseline in anxiety and/or sleep quality and decreases in acute medication use for migraine during fremanezumab treatment. Additionally, approximately three-quarters of patients surveyed reported that longer-term treatment with monthly or quarterly fremanezumab was associated with improvements in social interactions (quality and amount of time spent with family or friends, leisure activities, and performance at work or school). Patients also reported improvements from baseline in depressed mood during fremanezumab treatment. It should be noted that the results on improvement in depressed mood should be interpreted with caution, as these results are based on resurveyed patients to correct an error in wording of the original questionnaire.

The majority of patients (69%) preferred quarterly fremanezumab dosing, regardless of whether they received quarterly or monthly dosing during the extension study. As all patients received three injections in the first month of every 3-month cycle, the preference of more than two-thirds of patients for quarterly dosing suggests that the experience of receiving three subcutaneous injections in 1 day does not deter patients from preferring quarterly over monthly dosing. Furthermore, the majority of patients (> 75%) reported that they believed having dosing flexibility would make taking the migraine preventive treatment as prescribed easier and is something that they would value in a preventive treatment. Shared decision-making, wherein patient preferences are considered, has been shown to directly affect treatment adherence and, in turn, treatment success as patients are more likely to be compliant with a medication they helped select [20, 21]. Results from our study are in line with findings from a previous survey-based observational study of 417 adults with migraine [22]. In that study, patients reported that they were more likely to fill a prescription (63% to 77%) and remain adherent to treatment (62% to 80%) if they had the option of taking their preferred dosing schedule (monthly or quarterly) [22]. Together with the current study, these results support the utility of offering multiple, effective dosing options to patients with migraine and assessing patient preference between those dosing options.

In this study, fremanezumab was consistently preferred to prior preventive medications, primarily due to reductions in migraine frequency, pain intensity, and attack duration. This is in keeping with the results of a prior study of 250 migraine patients in which 72% of patients rated effectiveness as the most important aspect of headache prevention [23]. Similarly, separate survey-based studies demonstrated that migraine patients valued the efficacy (eg, 50% reduction in migraine days) of migraine preventive treatments more highly than safety or tolerability [24, 25]. While efficacy endpoints for both the placebo-controlled pivotal trials and the extension study focused primarily on migraine frequency, it is notable that patients in the current survey emphasized the value of fremanezumab for reducing the impact of remaining attacks by decreasing pain intensity and attack duration. This more holistic efficacy benefit of fremanezumab identified by patients appears to drive preference for this preventive treatment. Better understanding of the impact of fremanezumab across the three efficacy domains of frequency, severity, and duration may be worthwhile to more adequately assess the potential patient benefits of treatment.

Results of this survey may be limited by recall and participation bias since the survey was completed between 1 to 24 months after patients completed the extension trial. As noted in a recent analysis, relying on volunteers to respond to research inquiries may result in a non-representative sample of responders, as compared to non-responders. Despite these limitations, the sample in this survey study was representative of the overall treatment population, and results were similarly distributed regardless of headache frequency–based migraine diagnosis (CM or EM) or dosing regimen received (quarterly or monthly). The depressed mood item needed to be re-administered due to a wording error in the original survey, resulting in only a sub-sample providing data for this item. Additionally, although the survey study sample was representative of the overall trial patient population, selection bias may be present because participants who did not respond well in the parent study may have been less likely to enroll in the extension study or complete treatment in the extension study. Although the majority of patients completed the parent treatment studies, HALO EM and HALO CM (> 90% [16, 18]) and the 52-week extension study (> 75%), participation rates in the post hoc survey appear modest due, in part, to potential bias in the recruitment of study participants; investigator sites may have contacted patients who were judged to be amenable to the re-contact survey via email [26]. The actual number of patients who received and read the re-contact email is not known.

In this survey study, participants had completed up to 15 months of fremanezumab treatment. When asked in the survey to report treatment satisfaction with fremanezumab based on their recall, a high level of treatment satisfaction was reported overall and across all evaluated treatment dimensions in patients with CM and EM. Furthermore, more than 80% of patients preferred fremanezumab over their previous migraine preventive treatment. Additionally, more than half of migraine patients in the current study reported improvements from baseline in anxiety, depression, and/or sleep quality; decreases in acute medication use; and improvements across all quality-of-life domains for migraine during up to 15 months of fremanezumab treatment.

## Conclusions

The high satisfaction with fremanezumab, preference over prior treatments, and perceived value of dosing flexibility may result in patients being more likely to adhere to this migraine preventive treatment, thereby resulting in better clinical outcomes than traditional migraine preventive treatments. The high preference for quarterly over monthly dosing also suggests that fremanezumab, the only anti-CGRP mAb available with the option for home administration of quarterly dosing, may provide a unique patient-centric benefit for patients seeking the convenience of only four preventive treatment days per year.

## Supplementary information

**Additional file 1: Figure S1.** Study design for recruitment of survey population. **Figure S2.** Previously used migraine preventive medications by therapeutic category. **Figure S3.** Percentage of patients reporting satisfaction with different aspects of treatment and overall. **Figure S4.** Treatment satisfaction scores (on a 7-point scale) with fremanezumab and prior migraine preventive medications. **Figure S5.** Patients favoring fremanezumab injection experience or onabotulinumtoxinA injection experience. **Figure S6.** Patient-reported ratings for psychosocial and quality-of-life domains for all patients (*n* = 253). **Figure S7.** Proportion of EM and CM patients who reported improvements in psychosocial and quality-of-life domains. **Figure S8.** Patient-reported ratings for psychosocial and quality-of-life domains for EM and CM patients.

## Data Availability

Anonymized data, as described in this manuscript, will be shared upon request from any qualified investigator by the author investigators or Teva Pharmaceutical Industries, Ltd.

## Abbreviations

AMPP: American Migraine Prevalence and Prevention
mAbs: Monoclonal antibodies
CGRP: Calcitonin gene-related peptide
CM: Chronic migraine
EM: Episodic migraine
MAST: Migraine in America Symptoms and Treatment
SD: Standard deviation
SNRI: Serotonin-norepinephrine reuptake inhibitors
SSRI: Serotonin reuptake inhibitor
