# Supplementary material for: Improvements across a range of patient-reported domains with fremanezumab treatment: results from a patient survey study
Source: J Headache Pain. 2020 Sep 4;21(1):109. doi: 10.1186/s10194-020-01177-4 (PMC7487845; doi:10.1186/s10194-020-01177-4)
Supplement: Supplementary file 1 — Additional file 1: Figure S1. Study design for recruitment of survey population. Figure S2. Previously used migraine preventive medications by therapeutic category. Figure S3. Percentage of patients reporting satisfaction with different aspects of treatment and overall. Figure S4. Treatment satisfaction scores (on a 7-point scale) with fremanezumab and prior migraine preventive medications. Figure S5. Patients favoring fremanezumab injection experience or onabotulinumtoxinA injection experience. Figure S6. Patient-reported ratings for psychosocial and quality-of-life domains for all patients (n = 253). Figure S7. Proportion of EM and CM patients who reported improvements in psychosocial and quality-of-life domains. Figure S8. Patient-reported ratings for psychosocial and quality-of-life domains for EM and CM patients. [file 10194_2020_1177_MOESM1_ESM.docx]

**Additional file for:**

**Improvements Across a Range of Patient-reported Domains With Fremanezumab Treatment: Results From a Patient Survey Study**

Dawn C. Buse, Sanjay K. Gandhi, Joshua M. Cohen, Verena Ramirez-Campos, Blaine Cloud, Ronghua Yang, Robert P. Cowan

**Supplementary Figure 1. Study design for recruitment of survey population.**


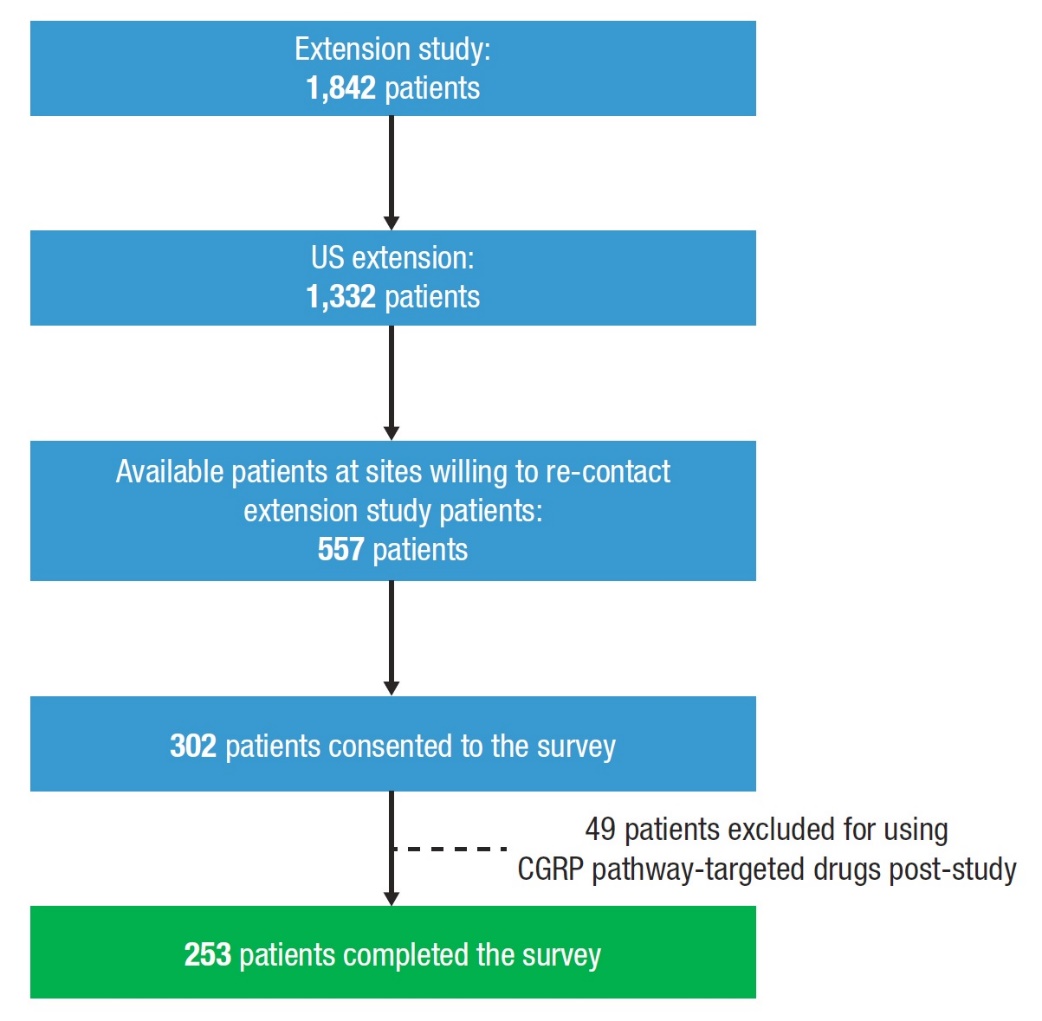


CGRP, calcitonin gene-related peptide.

**Supplementary Figure 2. Previously used migraine preventive medications by therapeutic category.**


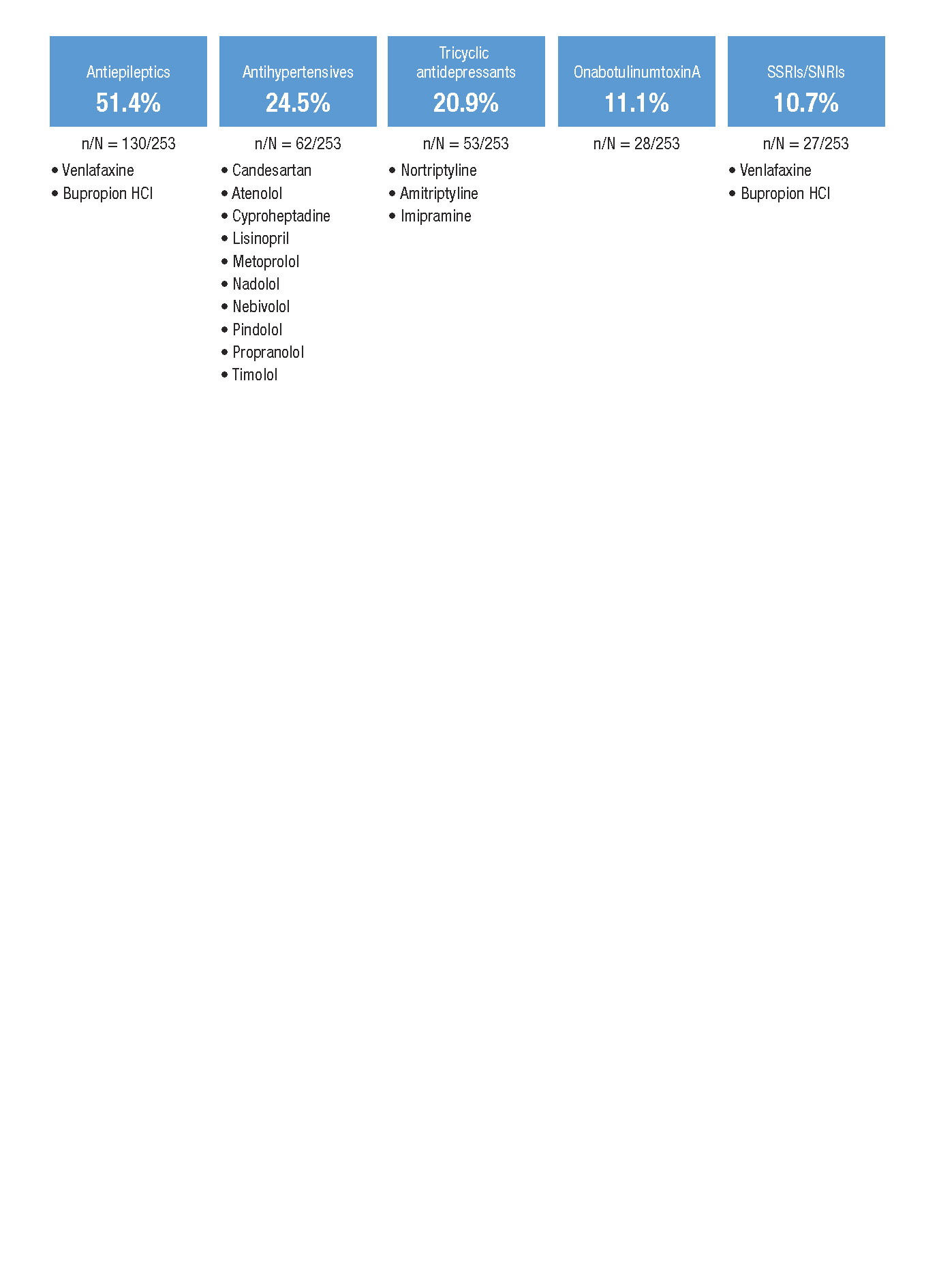


SNRI, serotonin-norepinephrine reuptake inhibitor; SSRI, selective serotonin reuptake inhibitor.

**Supplementary Figure 3. Percentage of patients reporting satisfaction with different aspects of treatment and overall.^a,b,c,d^**


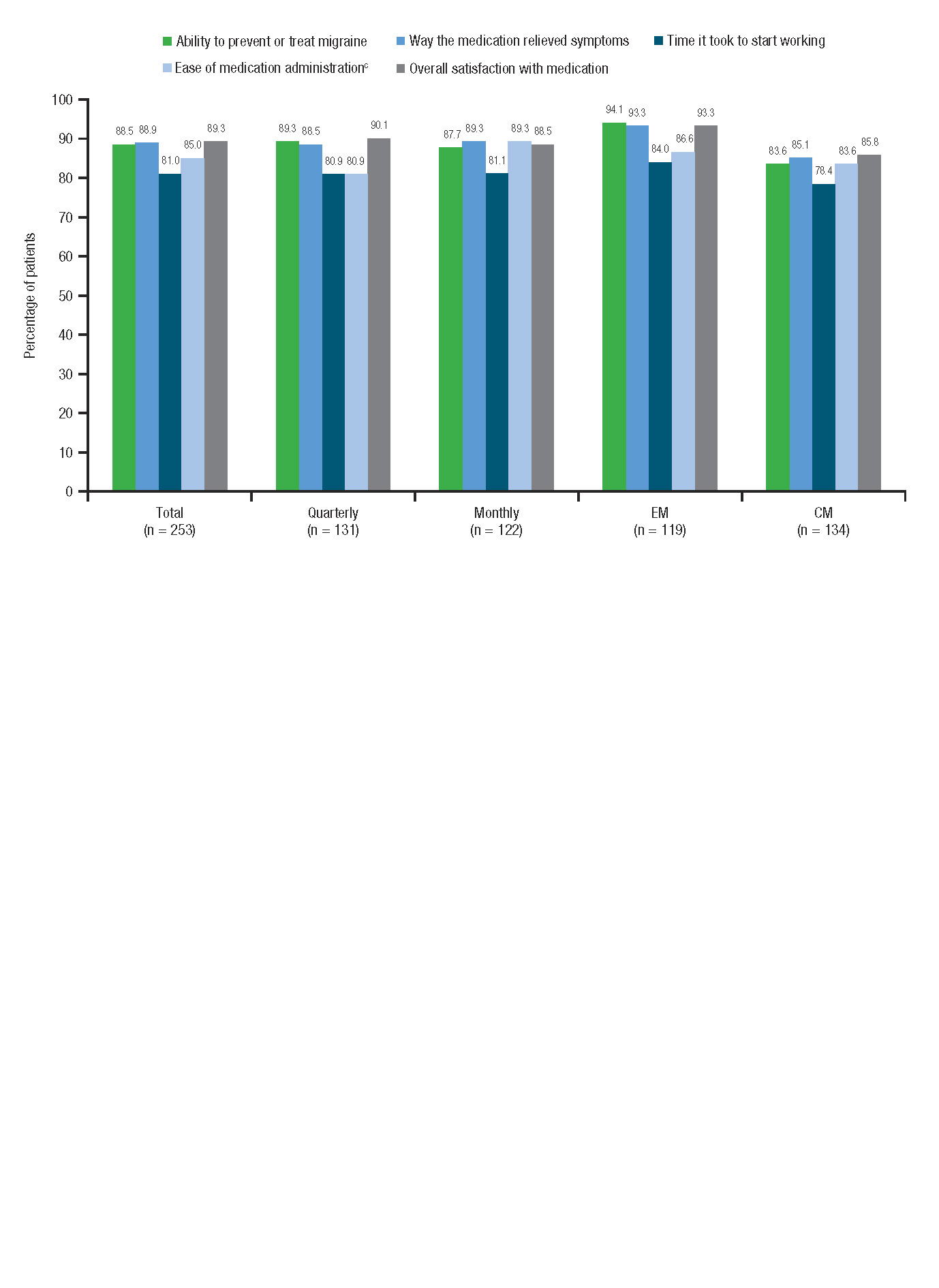


CM, chronic migraine; EM, episodic migraine.

^a^For ability to prevent or treat migraine, way the medication relieved symptoms, and time it took to start working, patients responded to the following questions: “How satisfied or dissatisfied were you with…”

- “…the ability of the clinical trial medication to prevent or treat your migraine?”
- “…the amount of time it took the clinical trial medication to start working?”
- “…the way the clinical trial medication relieved your migraine symptoms?”

^b^For ease of medication administration, patients responded to the following question: “How easy or difficult was it to have the clinical trial medication administered in the clinical trial site (doctor’s office)?”

^b^For overall satisfaction with medication, patients responded to the following question: “Taking all things into account, how satisfied or dissatisfied were you with the clinical trial medication?”

^d^The Likert Scale ranged from: 1 = “extremely dissatisfied” to 7 = “extremely satisfied”; or 1 = “extremely difficult” to 7 = “extremely easy” (for ease of medication administration question only). Ratings from 5 to 7 were included here.

**Supplementary Figure 4. Treatment satisfaction scores (on a 7-point scale) with fremanezumab and prior migraine preventive medications.^a,b^**

**
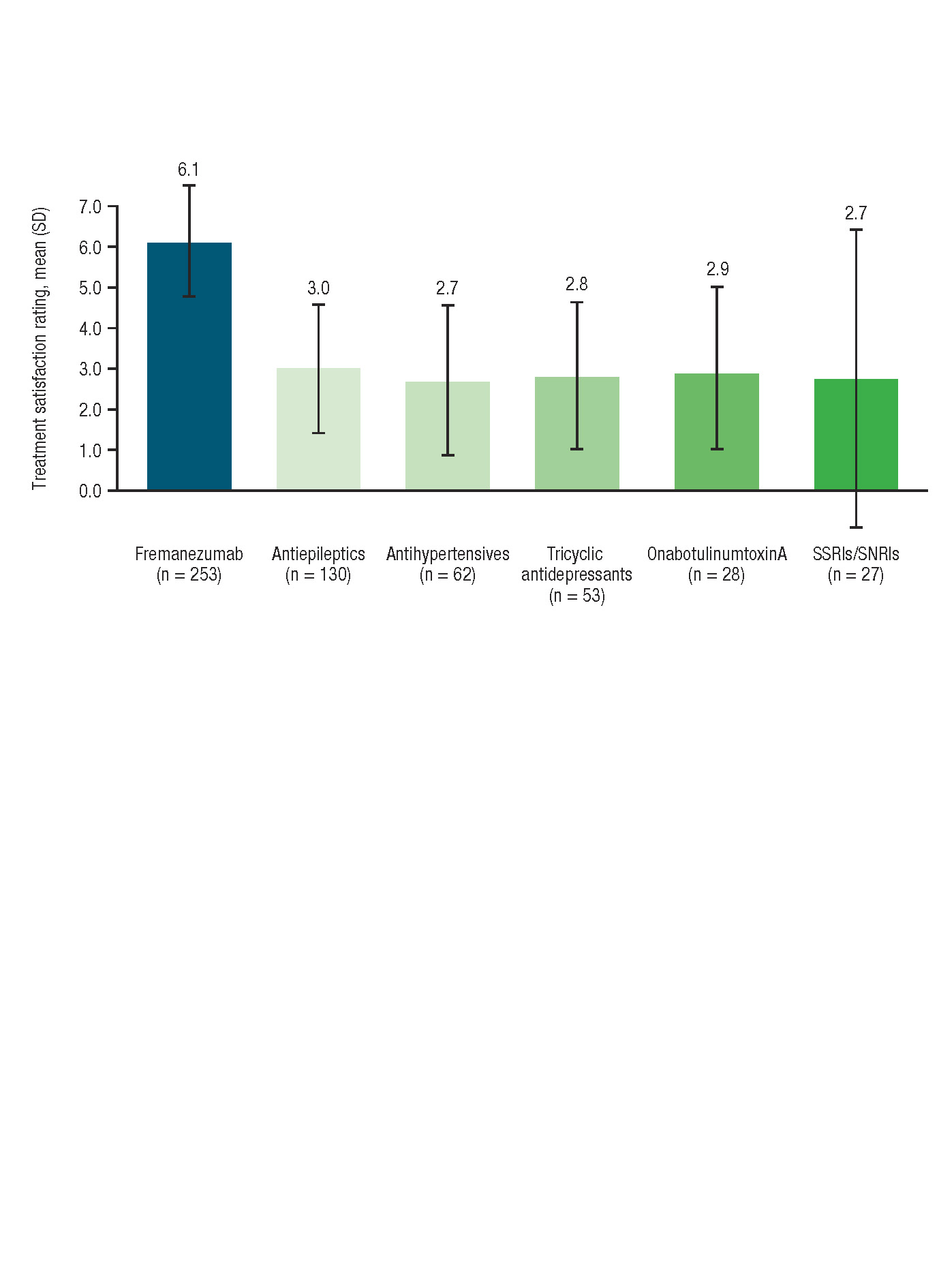
**

SD, standard deviation; SNRI, serotonin norepinephrine reuptake inhibitor; SSRI, selective serotonin reuptake inhibitor.

^a^For fremanezumab, patients responded to the following question: “How satisfied or dissatisfied were you with the ability of the clinical trial medication to prevent or treat your migraine?” Satisfaction was rated on a 7-point scale: 1 = “extremely dissatisfied” to 7 = “extremely satisfied.”

^b^For satisfaction with prior preventive medications, patients responded to the following question: “Using a 7-point scale where 1 means ‘extremely dissatisfied’ and 7 means ‘extremely satisfied,’ how satisfied or dissatisfied were you with the ability of (*prior migraine preventive medication*) to prevent or treat your migraine attacks?”

**Supplementary Figure 5. Patients favoring fremanezumab injection experience or onabotulinumtoxinA injection experience.^a^**


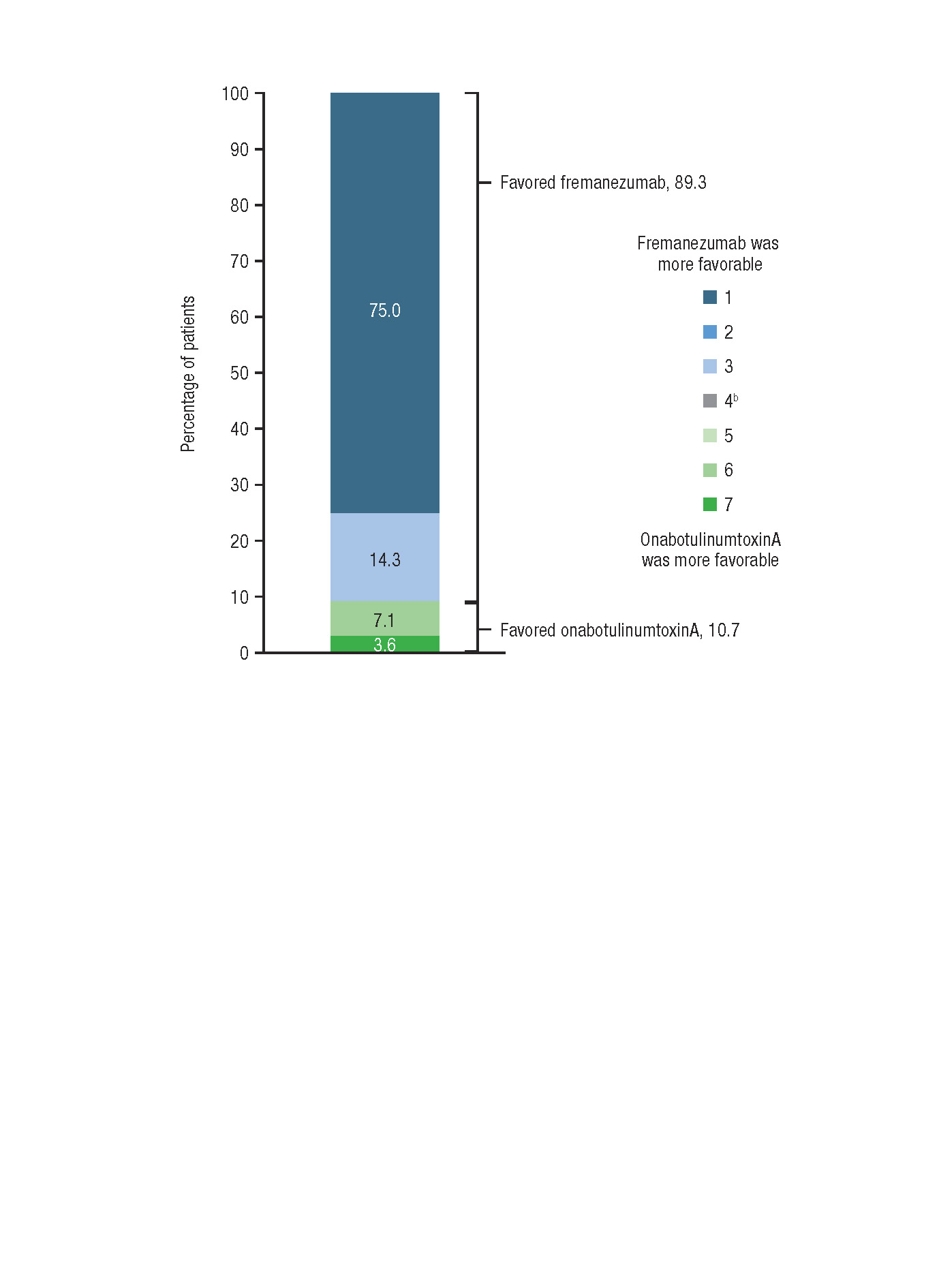


^a^Patients responded to the question: “You indicated that you’ve used onabotulinumtoxinA in the past. Thinking about your experience with the injections for the experimental medicine in the clinical trial and for onabotulinumtoxinA, which injection experience was more favorable? Please use a 7-point scale where 1 means ‘the experimental medicine’s injections were more favorable’ and 7 means ‘onabotulinumtoxinA injections were more favorable.’”

^b^Both injections had a similar experience.

**Supplementary Figure 6. Patient-reported ratings for psychosocial and quality-of-life domains for all patients (n = 253).^a,b,c,d^**

**
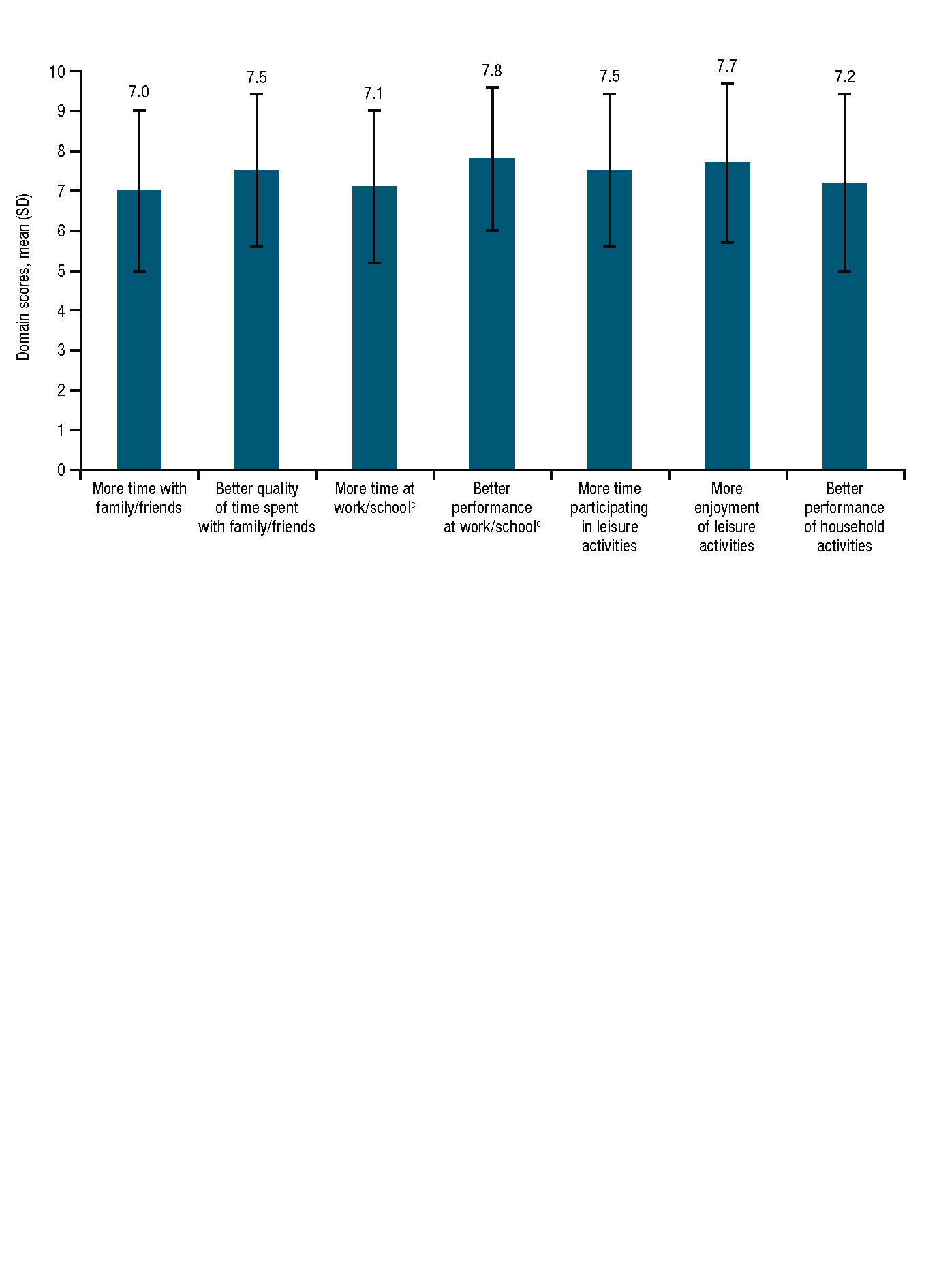
**

SD, standard deviation.

^a^Compared to baseline.

^b^For time with family/friends, quality of time with family/friends, time at work/school, time participating in leisure activities, and enjoyment of leisure activities, patients responded to the following questions: “Compared to the 3-month baseline period before the first injection, on a scale of 0 to 10, where 0 is ‘significantly less’ and 10 is ‘significantly more,’…”

- “…how much less or more time did you spend with friends and family while you were taking the study medicine?”
- “…how much change did you experience in the quality of time you spent with your friends and family while you were taking the study medicine?”
- “…how much less or more were you able to attend work or school while you were taking the study medicine?”
- “…how much less or more could you participate in leisure and personal activities (i.e., hobbies) while you were taking the study medicine?”
- “…how much less or more were you able to enjoy leisure and personal activities while you were taking the study medicine?”

^c^For performance at work/school and performance of household activities, patients responded to the following questions: “Compared to the 3-month baseline period before the first injection, on a scale of 0 to 10, where 0 is ‘significantly worse’ and 10 is ‘significantly better,’…”

- “…how did your **work or school performance** change while you were taking the study medicine?”
- “…how much did your ability to **perform household activities and chores** change while you were taking the study medicine?”

^d^n = 213.

**Supplementary Figure 7. Proportion of EM and CM patients who reported improvements in psychosocial and quality-of-life domains.^a,b,c^**


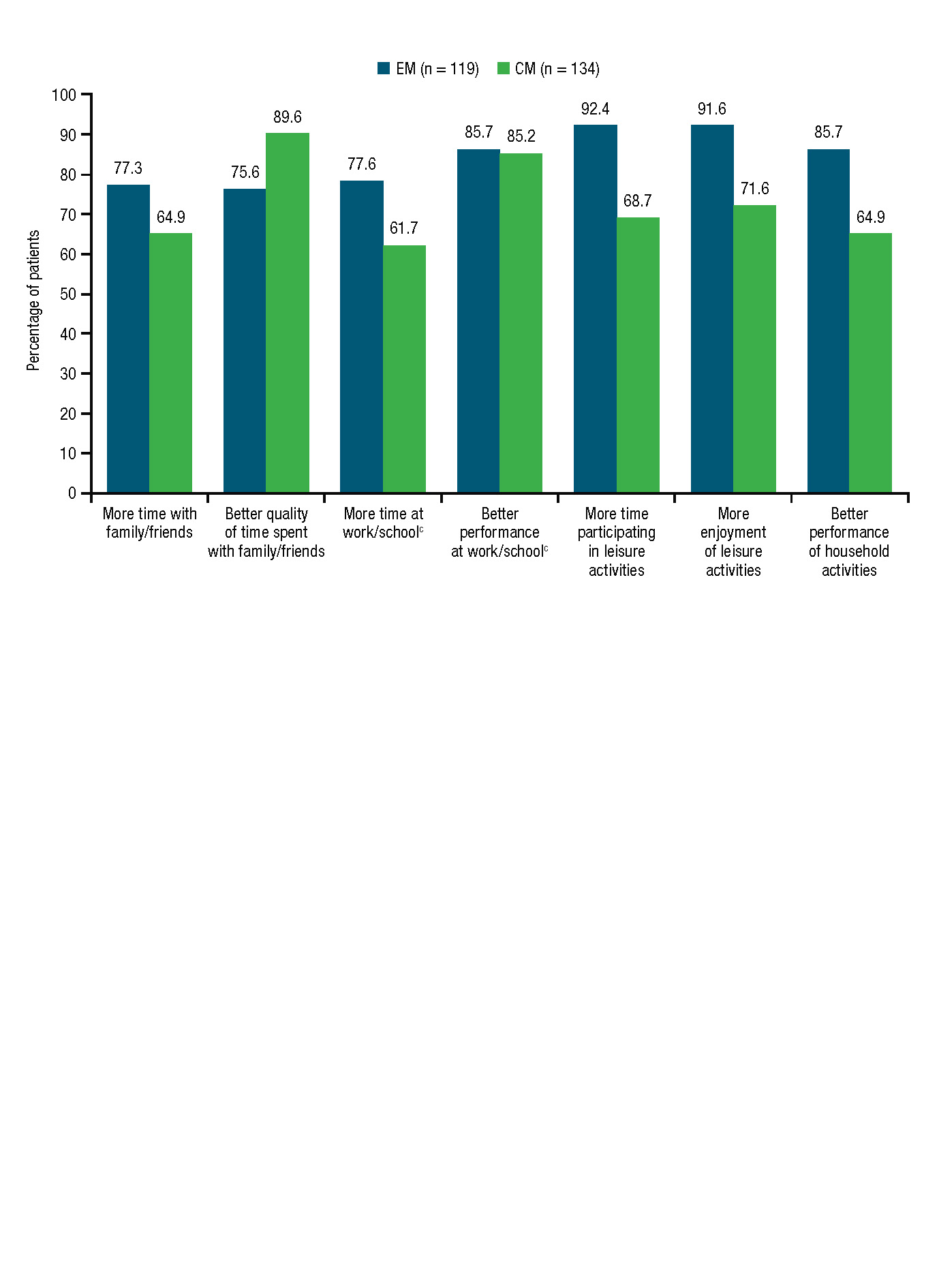


CM, chronic migraine; EM, episodic migraine.

^a^Compared to baseline.

^b^For time with family/friends, quality of time with family/friends, time at work/school, time participating in leisure activities, and enjoyment of leisure activities, patients responded to the following questions: “Compared to the 3-month baseline period before the first injection, on a scale of 0 to 10, where 0 is ‘significantly less’ and 10 is ‘significantly more,’…”

- “…how much less or more time did you spend with friends and family while you were taking the study medicine?”
- “…how much change did you experience in the quality of time you spent with your friends and family while you were taking the study medicine?”
- “…how much less or more were you able to attend work or school while you were taking the study medicine?”
- “…how much less or more could you participate in leisure and personal activities (i.e., hobbies) while you were taking the study medicine?”
- “…how much less or more were you able to enjoy leisure and personal activities while you were taking the study medicine?”

^c^For performance at work/school and performance of household activities, patients responded to the following questions: “Compared to the 3-month baseline period before the first injection, on a scale of 0 to 10, where 0 is ‘significantly worse’ and 10 is ‘significantly better,’…”

- “…how did your **work or school performance** change while you were taking the study medicine?”
- “…how much did your ability to **perform household activities and chores** change while you were taking the study medicine?”

^d^EM, n = 98; CM, n = 115.

**Supplementary Figure 8. Patient-reported ratings for psychosocial and quality-of-life domains for EM and CM patients.^a,b,c^**


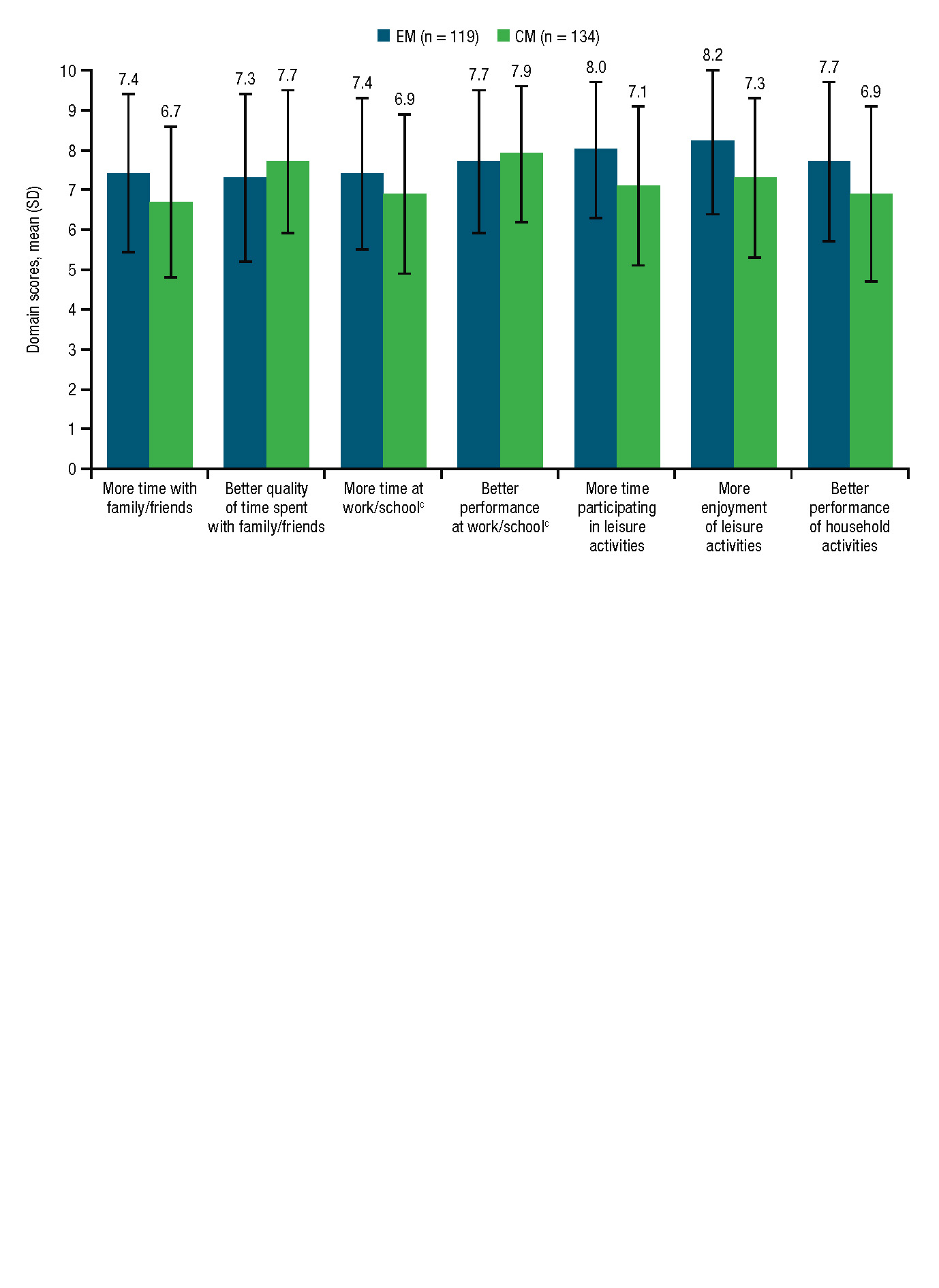


CM, chronic migraine; EM, episodic migraine; SD, standard deviation.

^a^Compared to baseline.

^b^For time with family/friends, quality of time with family/friends, time at work/school, time participating in leisure activities, and enjoyment of leisure activities, patients responded to the following questions: “Compared to the 3-month baseline period before the first injection, on a scale of 0 to 10, where 0 is ‘significantly less’ and 10 is ‘significantly more,’…”

- “…how much less or more time did you spend with friends and family while you were taking the study medicine?”
- “…how much change did you experience in the quality of time you spent with your friends and family while you were taking the study medicine?”
- “…how much less or more were you able to attend work or school while you were taking the study medicine?”
- “…how much less or more could you participate in leisure and personal activities (i.e., hobbies) while you were taking the study medicine?”
- “…how much less or more were you able to enjoy leisure and personal activities while you were taking the study medicine?”

^c^For performance at work/school and performance of household activities, patients responded to the following questions: “Compared to the 3-month baseline period before the first injection, on a scale of 0 to 10, where 0 is ‘significantly worse’ and 10 is ‘significantly better,’…”

- “…how did your **work or school performance** change while you were taking the study medicine?”
- “…how much did your ability to **perform household activities and chores** change while you were taking the study medicine?”

^d^n = 213.
